# Green Tea Extract (*Theaceae*; *Camellia sinensis* L.): A Promising Antimicrobial, Anti-Quorum Sensing and Antibiofilm Candidate Against Multidrug-Resistant *Campylobacter* Species

**DOI:** 10.3390/antibiotics14010061

**Published:** 2025-01-09

**Authors:** Mona S. Emara, Ahmed M. Ammar, Ashraf M.O. Abdelwahab, Attia A. Elgdawy, Adel Abdelkhalek, Elena Pet, Gabi Dumitrescu, Mirela Ahmadi, Norhan K. Abd El-Aziz

**Affiliations:** 1 Animal Health Research Institute, Zagazig 44516, Egypt; dr.mona.samir.emara@ahri.gov.eg; 2 Department of Microbiology, Faculty of Veterinary Medicine, Zagazig University, Zagazig 44511, Egypt; amamar@vet.zu.edu.eg (A.M.A.);; 3 Animal Health Research Institute, Giza 12618, Egypt; dr.attia31@yahoo.com; 4 Food Safety, Hygiene and Technology Department, Faculty of Veterinary Medicine, Badr University in Cairo (BUC), Badr City 11829, Egypt; adel.abdelkhalek@buc.edu.eg; 5 Faculty of Management and Rural Tourism, University of Life Sciences “King Mihai I” from Timisoara, Calea Aradului 119, 300645 Timisoara, Romania; 6 Faculty of Bioengineering of Animal Resources, University of Life Sciences “King Mihai I” from Timisoara, Calea Aradului 119, 300645 Timisoara, Romania; gdumitrescu@animalsci-tm.ro (G.D.); mirelaahmadi@usvt.ro (M.A.)

**Keywords:** *Campylobacter* species, antimicrobial resistance, biofilm formation, herbal extracts

## Abstract

Background: Thermophilic *Campylobacter* species are among the main culprits behind bacterial gastroenteritis globally and have grown progressively resistant to clinically important antimicrobials. Many studies have been carried out to explore innovative and alternative strategies to control antibiotic-resistant campylobacters in animal reservoirs and human hosts; however, limited studies have been performed to develop efficient control schemes against *Campylobacter* biofilms. Methods: This study investigated the antimicrobial and antibiofilm activities of some herbal extracts against multidrug-resistant (MDR) *Campylobacter* species recovered from different sources using phenotypic and molecular techniques. Results: The overall *Campylobacter* species prevalence was 21.5%, representing 15.25% and 6.25% for *C. jejuni* and *C. coli*, respectively. Regarding *C. jejuni*, the highest resistance rate was observed for amoxicillin–clavulanic acid and colistin (85.25% each), followed by cefotaxime (83.61%) and tetracycline (81.97%), whereas *C. coli* isolates showed absolute resistance to cefotaxime followed by erythromycin (92%) and colistin (88%). Remarkably, all *Campylobacter* isolates were MDR with elevated multiple antimicrobial resistance (MAR) indices (0.54–1). The antimicrobial potentials of green tea (*Camellia sinensis*), rosemary (*Rosmarinus officinalis*) and ginger (*Zingiber officinale*) extracts against MDR *Campylobacter* isolates were assessed by the disk diffusion assay and broth microdilution technique. Green tea extract showed a marked inhibitory effect against tested isolates, exhibiting growth inhibition zone diameters of 8 to 38 mm and a minimum inhibitory concentration (MIC) range of 1.56–3.12 mg/mL, unlike the rosemary and ginger extracts. Our findings reveal a respectable antibiofilm activity (>50% biofilm formation inhibition) of green tea against the preformed biofilms of *Campylobacter* isolates. Furthermore, real-time quantitative polymerase chain reaction (RT-qPCR) results showed a significant decrease (*p* < 0.05) in the expression levels of biofilm biosynthesis gene and its regulator (*FlaA* and *LuxS*, respectively) in *Campylobacter* isolates treated with the green tea extract in comparison with untreated ones. Conclusion: This is the first in vitro approach that has documented the inhibitory activity of green tea extract against MDR-biofilm-producing *Campylobacter* species isolated from different sources. Further in vivo studies in animals’ models should be performed to provide evidence of concept for the implementation of this alternative candidate for the mitigation of MDR *Campylobacter* infections in the future.

## 1. Introduction

Campylobacteriosis is a zoonotic illness caused by *Campylobacter* species; these have been reported as the main foodborne pathogens and the most common bacterial causes of gastroenteritis in humans worldwide [1]. *Campylobacter* is a gram-negative bacilli, spiral or slightly curved, motile, non-spore forming and microaerophilic bacteria [2]. *Campylobacter jejuni* (*C. jejuni*) and *Campylobacter coli* (*C. coli*) are the two major species responsible for severe cases of human gastroenteritis [3].

*Campylobacter* species may exist in aggregated communities enclosed in a self-produced matrix known as a biofilm [4]. The molecular basis of biofilm development in *Campylobacter* is still not fully understood, although there is evidence that quorum sensing represented by S-ribosylhomocysteine lyase (*luxS*), flagella, and surface proteins, are necessary to optimize biofilm formation. Several studies have demonstrated that the *luxS* gene is implied in various physiological pathways in *C. jejuni*, including motility, flagellar expression, oxidative stress, autoagglutination, and animal colonization [5,6,7,8]. The biofilm formation results in increased resistance to negative environmental influences, including resistance to antimicrobial agents, resulting in chronic infections [9]. Treatment of campylobacteriosis may be complicated by the emergence of multidrug-resistant (MDR) and extensively drug-resistant (XDR) *Campylobacter* species, which may be attributed to the excessive and uncontrolled use of antimicrobials in the poultry industry, livestock farming and veterinary medicine [10,11]. The ongoing spread and emergence of drug-resistant *Campylobacter* species exacerbate therapeutic challenges, raising the frequency of diseases and fatalities.

In the literature, green tea (*Camellia sinensis*), rosemary (*Rosmarinus officinalis*) and ginger (*Zingiber officinale*) extracts have been shown to exhibit resonant antimicrobial activities against pathogenic gram-negative bacteria and thus may support the development of antimicrobial supplements [12,13,14]. Green tea is rich in polyphenols termed catechins, especially epigallocatechin gallate (EGCG) which is a major antimicrobial compound against various pathogenic organisms [15]. These catechins showed high activity against gram-negative and gram-positive bacteria using several molecular mechanisms, including the inhibition of the cell membrane, cell wall, nucleic acid and protein syntheses, and the inhibition of various metabolic pathways, such as extracellular matrix virulence factors, toxins, iron chelation, and oxidative stress. Rosemary extract is a household herb that comprehends a number of phytochemicals, comprising rosmarinic acid, caffeic acid, camphor, betulinic acid, ursolic acid, and the antioxidant carnosic acid [16]. Rosemary leaf extracts have a variety of in vitro bioactivities including antioxidant, antibacterial, anti-tumor, antiulcerogenic, antidiuretic, antidiabetic, anti-inflammatory and antithrombotic activities [17]. Additionally, ginger extract is an excellent source of a variety of bioactive composites, including bioactive phenols (shogaols, gingerols, and zingerones) [18]. Thus, more studies pertaining to the use of plant extracts as therapeutic agents should be emphasized, especially those related to the control of antibiotic-resistant microbes. The objective of this study is to investigate the antimicrobial, antibiofilm and anti-quorum sensing activities of plant-derived natural products as promising contenders of sustainable alternatives for combating drug resistance and treatment of biofilm-associated infections.

## 2. Results

### 2.1. Prevalence of Campylobacter Species in Animal, Environmental and Human Sources

As illustrated in Table 1, out of 290 chicken samples, 66 (22.76%) *Campylobacter* isolates were recovered, represented as 15.52% *C. jejuni* and 7.24% *C. coli*. However, examination of environmental samples revealed only *C. jejuni* with a percentage of 8.33%. Regarding human samples, *Campylobacter* species was detected in 30% of cases, with 20% being *C. jejuni* and 10% *C. coli*. Statistical analysis showed significant disparities in the prevalence of *Campylobacter* isolates among chickens and their products, environmental sources, and human stool (*p* = 0.0137). Noteworthy variations were also found within each source category, especially between chickens and their products, with the highest rates appearing in liver samples (60.00%). Conversely, no significant distinctions were observed in the occurrence of these microbial isolates across different environmental sources.

Conventional identification of *Campylobacter* species was simply determined. On mCCDA agar, *C. jejuni* appeared as grey and moist spreading colonies, whereas *C. coli* was creamy-grey with slightly raised discrete colonies. Biochemical characteristics of campylobacters yielded positive results for catalase, oxidase, and nitrate reduction tests. All isolates were sensitive to nalidixic acid and resistant to cephalothin. However, at the species level, *C. jejuni* isolates were hippurate and indoxyl acetate positive, while *C. coli* isolates were indoxyl acetate positive and hippurate negative. Molecular confirmation of the recovered isolates was performed based on the genus-specific (*23S rRNA*) and species-specific (*mapA*, and *ceuE*) primers, giving characteristic bands at 650, 589 and 462 bps for *Camplobacter* species, *C. jejuni* and *C. coli*, respectively.

### 2.2. Antimicrobial Susceptibility Testing of Campylobacter Species

**Figure 1:**
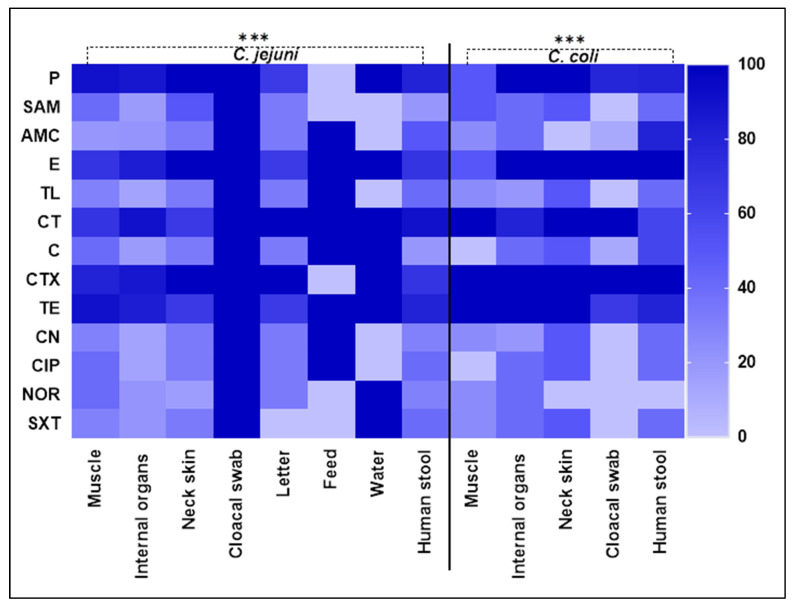
The heatmap illustrates the antimicrobial resistance of *Campylobacter* species recovered from different sources. Dark colors refer to the antimicrobials displaying high resistance levels and light colors refers to the antimicrobials displaying low resistance. Color bar on the right side indicates color intensity. AMA, antimicrobial agent; P, penicillin; SAM, ampicillin–sulbactam; AMC, amoxicillin–clavulanic acid; E, erythromycin; TL, tylosin; CT, colistin; C, chloramphenicol; CTX, cefotaxime; TE, tetracycline; CN, gentamycin; CIP, ciprofloxacin; NOR, norfloxacin; SXT, sulfamethoxazole–trimethoprim. *** indicate significant differences at *p* < 0.05.

The antibiogram of *Campylobacter* isolates (n = 86) comprising 61 *C. jejuni* and 25 *C. coli* against 13 tested antimicrobial agents is presented in Table 2 and in Figure 1. Regarding *C. jejuni* isolates, the greatest rate of resistance was noted for penicillin and colistin (85.25% each), followed by cefotaxime (83.61%) and tetracycline (81.97%), whereas gentamycin showed the least resistance rate (22.95%), followed by ampicillin–sulbactam and tylosin (26.23% each), chloramphenicol, ciprofloxacin, norfloxacin, and sulfamethoxazole–trimethoprim (27.87% each) and amoxicillin–clavulanic acid (29.51%). On the other hand, *C. coli* isolates showed absolute resistance to cefotaxime, followed by erythromycin (92%) and colistin (88%). Interestingly, all *Campylobacter* isolates were found to be MDR, exhibiting resistance to 8 to 13 antimicrobial agents. The antimicrobial resistance of *C. jejuni* isolates exhibited significant differences across all studied antimicrobials, with the exception of erythromycin, colistin, and tetracycline (*p* = 0.5416, 0.6129, and 0.4534, respectively; Table 2). In the case of *C. coli*, significant differences were observed against all examined antimicrobials, except for the three mentioned earlier (*p* = 0.5153, 0.7641, 1.00, and 0.5686, respectively; Table 2).

### 2.3. Biofilm Formation by Multidrug-Resistant Campylobacter Isolates

The antimicrobial resistance patterns and the degrees of biofilm formation in 20 MDR *Campylobacter* isolates (16 *C. jejuni* and 4 *C. coli*) are presented in Table 3. The MDR *Campylobacter* isolates exhibiting high MAR indices (0.54–1) were examined for biofilm production using the microtiter plate method. The results reveal that tested isolates differ in their ability to form biofilms. All examined *Campylobacter* isolates of human origin (n = 3) were strong biofilm producers, whereas 8 (57.1%), 5 (35%) and 1 (7.11%) out of 14 *Campylobacter* isolates of chicken origin were strong, moderate, and weak biofilm producers, respectively. Moreover, the three isolates of environmental source were strong biofilm producers.

### 2.4. Antimicrobial Activities of Green Tea, Rosemary and Ginger Extracts Against MDR Campylobacter Species

As shown in Table 4, all MDR *Campylobacter* isolates were sensitive to the green tea extract, exhibiting zones of growth inhibition ranging from 8 to 38 mm and MIC values of 1.56–3.12 mg/mL. Meanwhile, MDR *Campylobacter* isolates were inhibited by rosemary extract at higher concentrations (MIC range of 25–50 mg/mL). On the other hand, MDR *Campylobacter* isolates showed modest sensitivity to the ginger extract, exhibiting growth inhibition zones ranging from 6 to 20 mm and an MIC range of 12.5–50 mg/mL. The negative control phosphate buffer saline (PBS) revealed no inhibition zones for all examined isolates. The impact of various concentrations of plant extracts on *C. jejuni* and *C. coli* exhibited noteworthy variances (*p* < 0.05). Regression analysis for the disc diffusion unveiled that the zone diameters decreased by 0.576 mm, 0.334 mm, and 0.370 mm with every unit decrease in concentrations of green tea, rosemary, and ginger extracts, respectively (*p* < 0.05). Moreover, the MIC values significantly differed, notably being lower with green tea compared with rosemary and ginger extracts.

### 2.5. Characterization of Green Tea Extract Using HPLC

Analysis of green tea extract revealed eight characteristic components; the gallic acid was the most abundant constituent (424.69 µg/mL) followed by rutin (176.50 µg/mL) and catechin (150.77 µg/mL). Various constituents were not detected in the study extract when compared with the standard green tea, as presented in Table 5.

### 2.6. Analysis of Green Tea Extract Using Liquid Chromatography–Electrospray Ionization–Tandem Mass Spectrometry (LC-ESI-MS/MS)

#### 2.6.1. Total Phenolic and Flavonoid Contents of Green Tea

**Figure 2:**
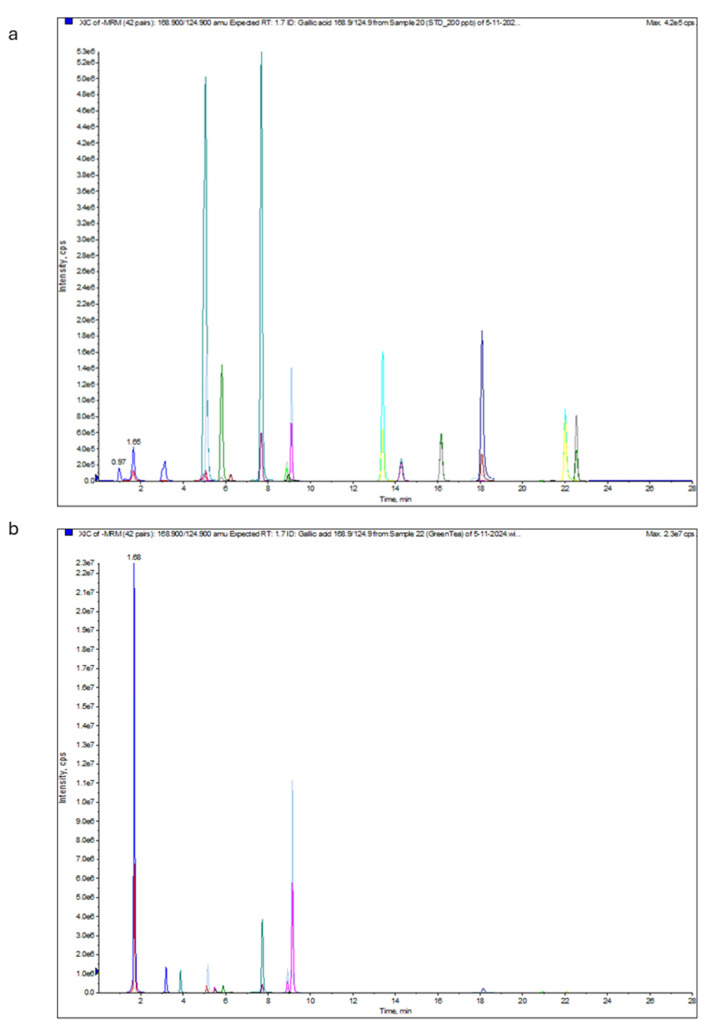
LC-ESI-MS/MS multiple reaction monitoring (MRM) chromatogram of the standard sample (**a**) and green tea extract sample (**b**). Different colours indicate extract constituents, and each analyte has two fragments generated at the same extension time. The scientific notation (i.e., 1.0e+6 is equal to 1 × 10^6^) could be converted into decimal notation using the following website: https://converthere.com/numbers/2.3e+7-written-out (accessed on 2 December 2024).

The total phenolic and flavonoid contents of green tea extract are shown in Table 6 and Figure 2. LC-ESI-MS/MS could identify 20 phenolic and flavonoid constituents in the analyzed green tea sample. Gallic acid was the most abundant at the negative mode with a concentration of 86.51 µg/mL, followed by naringenin (47.01 µg/mL). Peak identification was based on analysis of the obtained data and direct comparison with standards.

#### 2.6.2. Catechins of Green Tea Extract

**Figure 3:**
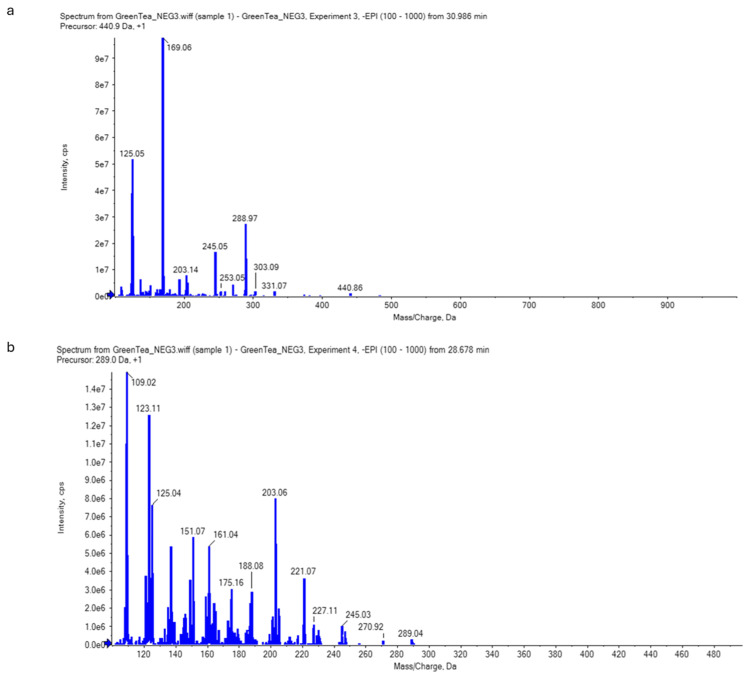
Catechins (**a**) and epigallocatechin gallate [M-H2O-H]- (EGCG) (**b**) identified in the green tea extract by LC-ESI-MS/MS in negative mode. The scientific notation (i.e., 1.0e6 is equal to 1 × 10^6^) could be converted into decimal notation using the following website: https://converthere.com/numbers/2.3e+7-written-out (accessed on 2 December 2024).

**Figure 4:**
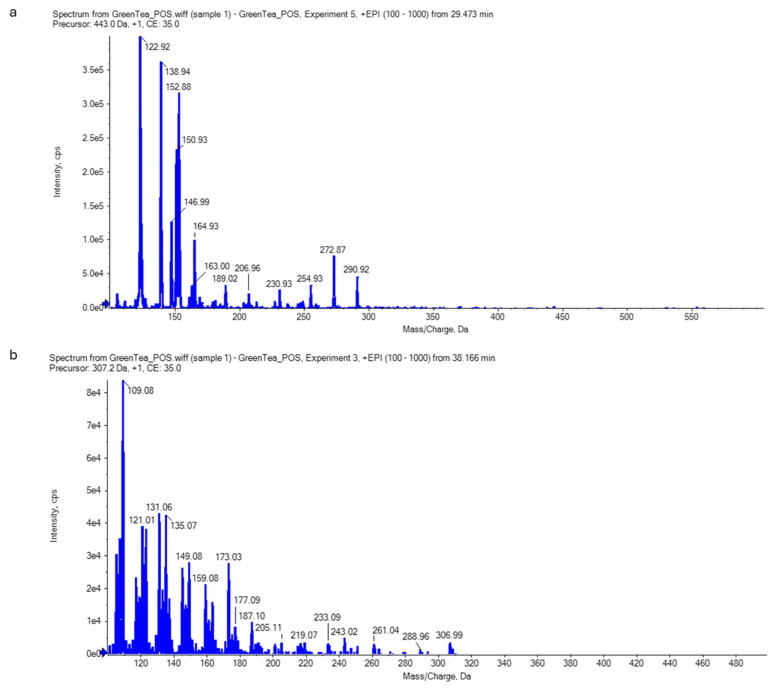
Epicatechin–gallate (ECG) (**a**) and epigallocatechine (EGC) (**b**) identified in the green tea extract by LC-ESI-MS/MS in positive mode. The scientific notation (i.e., 1e4 is equal to 1 × 10^4^) could be converted into decimal notation using the following website: https://converthere.com/numbers/2.3e+7-written-out (accessed on 2 December 2024).

The LC-ESI-MS/MS could identify the main secondary metabolites of green tea, especially of catechins, using the retention time and mass spectral information. The results were found to reveal epigallocatechin gallate, EGCG [M-H2O-H]- at the negative mode (Figure 3) and both epicatechin–gallate (ECG) and epigallocatechine (EGC) at the positive mode (Figure 4).

### 2.7. Antibiofilm Activity of Green Tea Extract Against Campylobacter Species

The antibiofilm activity of green tea extract against pre-existing biofilms produced by *C. jejuni* and *C. coli* isolates appeared to be concentration dependent (1.56–3.12 mg/L). Our results show good antibiofilm activity (>50% inhibition of biofilm formation) of green tea extract against the preformed biofilms of *Campylobacter* isolates (Table 7). The results were compared with those of untreated *C. jejuni* and *C. coli* biofilm producers (positive control) as well as non-biofilm producers (negative control). Statistical analysis revealed that the antibiofilm activity values of green tea extract against MDR biofilm-producing *Campylobacter* isolates were markedly greater pre-treatment compared with post-treatment, with a significant difference noted (*p* = 0.0001).

### 2.8. Transcriptional Changes of Biofilm Genes Post Treatment by Green Tea Extract

**Figure 5:**
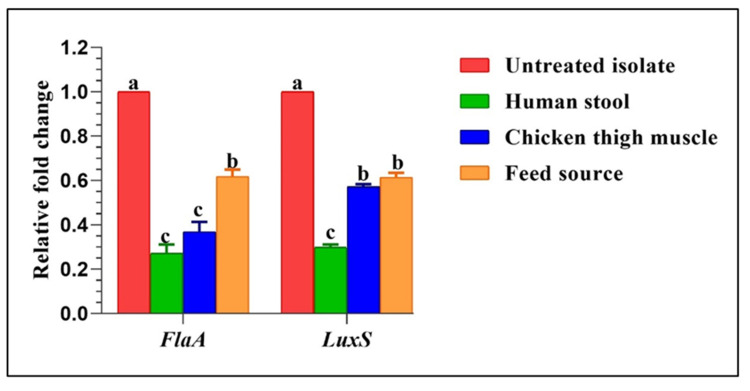
Fold changes in the expression levels of examined biofilm genes after treatment of *Campylobacter* isolates with sub-inhibitory concentrations of green tea extract. a–c Values of the same row with different superscripts are significantly different (*p* < 0.05).

To further confirm the subinhibitory concentration (SIC) effects of green tea extract on pre-existing biofilms of the strong biofilm-producing *Campylobacter* isolates, transcript levels of the expression of the biofilm-associated genes, *FlaA* and *LuxS*, were evaluated by RT-qPCR (Figure 5). In all examined isolates, the transcription levels of both genes were notably decreased in comparison to the untreated isolates (*p* < 0.05), which are assigned a value of 1.0, minimizing in human stool (changes of up to 0.2432 and 0.3078-fold for the abovementioned genes, respectively), which illustrated the strong antibiofilm activity of the green tea against MDR strong biofilm-producing *Campylobacter* isolates. No significant variances were found between *Campylobacter* isolates recovered from human stool and chicken thigh muscles concerning the *FlaA* gene expression, as well as between those obtained from the environmental source (feed) and chicken thigh muscles regarding the *LuxS* gene expression (*p* > 0.05).

## 3. Discussion

Thermotolerant campylobacters, predominantly *C. coli* and *C. jejuni*, are considered the most important agents of bacterial gastroenteritis worldwide. Currently, an emerging problem among campylobacters is the elevating resistance to major antibiotics in use [19]. Hence, this study investigated the antimicrobial and antibiofilm activities of some plant-derived extracts against MDR biofilm-producing *Campylobacter* isolates.

Herein, the prevalence of *Campylobacter* species in chicken samples was 22.76%, which was lower than a previous study in Egypt (58.11%) [19]. However, a slightly higher prevalence rate of *Campylobacter* species has been previously documented in the united states (25.4%) [20]. It is known that poultry can be contaminated from a variety of sources on farms and that the contaminants are dispersed through processing, scalding, defeathering, evisceration and giblet operations; further spread can occur during handling in markets and kitchens [21]. On the other hand, examination of environmental samples revealed only *C. jejuni,* with a percentage of 8.33%, which was lower than a previous record in Egypt (13.3%) [22]. Regarding human samples, *Campylobacter* species was detected in 30% of examined stool samples, which was lower than previous studies at 14.7% [22], 26% [23] and 83.33% [24], but higher than another report in Egypt (2.7%) [25]. Variations in the prevalence of *Campylobacter* species across different studies can be attributed to numerous factors, such as bacterial contamination, health conditions, geographical locations, climate influences, sources of the samples analyzed, and the traditional methods used for identification [26].

Lately, resistance of *Campylobacter* species to antimicrobial agents has become an important public health problem around the world, particularly resistance to fluoroquinolones, macrolides, tetracycline, aminoglycoside and beta lactams [27]. Furthermore, intrinsic resistance has been described in *C. jejuni* and *C. coli* against penicillin, most cephalosporins, trimethoprim sulfamethoxazole, vancomycin and rifampicin [28,29,30]. Interestingly, all *Campylobacter* isolates in this study were MDR, exhibiting resistance to 8 to 13 antimicrobial agents, which is similar to the results of a previous study in China [31]. Unsurprisingly, there is variation in antimicrobial resistance between and within different countries, which is closely related to the types of drugs prescribed as well as variation in guidelines for the use of antimicrobial drugs. These findings are alarming and correlate well with the uncontrolled use of drugs as growth promotors and prophylaxes in animal production [32,33,34]. Hence, this study highlighted the necessity of using antimicrobial agents sparingly in veterinary medicine to avoid the emergence of antimicrobial resistance in both humans and animals. Furthermore, it is highly recommended to look for novel, appropriate, and potent natural antimicrobial compounds, especially against *Campylobacter* infections.

Medicinal plants possess remarkable potential for generating a wide array of bioactive molecules with antibacterial properties, offering a vast and inexhaustible repertoire [35,36,37,38]. Moreover, they interact safely with the body’s vital systems, exhibiting minimal side effects [39]. In the current study, we evaluated the antimicrobial activities of green tea, rosemary and ginger extracts against MDR *Campylobacter* species. Our findings proved that green tea extract demonstrated strong antimicrobial activity against examined isolates, whereas rosemary and ginger extracts exhibited only moderate to weak antimicrobial effects.

The important components of green tea that show antimicrobial properties are the catechins. The four main catechins in green tea are epicatechin (EC), epicatechin-3-gallate (ECG), epigallocatechin (EGC), and epigallocatechin-3-gallate (EGCG). Of these catechins, EGCG and EGC are abundant and have been shown to demonstrate antimicrobial properties against various bacterial species [40]. Upon reviewing the literature, numerous natural compounds have been used to combat a variety of microbial genera and/or species [41,42,43]. However, there were no reports found to be available on the antimicrobial activity of green tea against *Campylobacter* species, at least in Egypt. However, previous studies have focused on the inhibitory activity of green tea on *E. coli*, *S.* Typhi and *S.* Typhimurium [44].

On the other hand, Friedman and coworkers [45] have demonstrated that ginger extract exhibits moderate antimicrobial effect against *Campylobacter* species, whereas Mutlu-Ingok et al. [46] have reported the low activity of the abovementioned extract against corresponding isolates. The active ingredients in the ginger extract, including phenyl propanoid-derived compounds, particularly gingerols, paradol, shogaols, and zingerone, are responsible for its antimicrobial activity against certain pathogens [47]. However, the inhibitory activity of rosemary oil against *Campylobacter* species may be attributed to the inclusion of rosmarinic acid, carnosic acid and their derivatives, such as carnosonal, rosmanol and isorosmanol. These substances interact with bacteria cell membranes to modify their genetic materials, electron transport, cellular component leakage, and fatty acid synthesis [48,49]. It has been documented that the effectiveness of carsonic acid against pathogenic bacteria exceeds that of other major extract components, including rosmarinic acid [50].

As the green tea extract exerted potent antimicrobial activity against the *Campylobacter* species under study, it was characterized using high-performance liquid chromatography (HPLC) and liquid chromatography–electrospray ionization–tandem mass spectrometry (LC-ESI-MS/MS). As demonstrated in the HPLC analysis datasheet of green tea extract, gallic acid presented the most abundant constituent. Gallic acid is a natural component in many traditional Chinese medications, and has antibacterial, and antiseptic activities. Gallic acid exerted a bactericidal effect against MDR *E. coli* via its disruption of the integrity of the outer and inner membranes and by suppressing the expression of efflux pump genes. Thus, gallic acid holds promise as a possible bactericidal agent with which to combat the emergence and spread of MDR *E. coli* [51]. Similarly, LC-ESI-MS/MS analysis revealed that gallic acid was the most abundant ingredient at the negative mode, with a concentration of 86.51 µg/mL. Moreover, EGCG, ECG and EGC could be separated and have been documented previously to demonstrate strong antimicrobial activities [52].

Biofilms are microbial communities that develop on surfaces or interfaces (e.g., air and liquid) and are embedded in a three-dimensional matrix of self-produced extracellular polymeric substances (EPS), primarily including exopolysaccharides, nucleic acids, and proteins [53]. Their development involves the initial attachment of planktonic (free-swimming) microorganisms to a surface, followed by proliferation and EPS production, microcolony formation, maturation (i.e., development of a characteristic biofilm architecture), and detachment. Within biofilms, microorganisms are protected against the antimicrobial activities of various substances, including well-established antibiotics [54]. Herein, the green tea extract exhibited good antibiofilm activity (>50% inhibition of biofilm formation) against the performed biofilms of *Campylobacter* isolates. This may be attributed to the EGCG component of the analyzed extract, which has previously been shown to have antibiofilm activities against bacterial biofilms [55].

The molecular ways by which biofilms shield bacteria from antimicrobial action are multifactorial. The EPS structure hinders the penetration of specific antibiotics and can contain enzymes that actively inactivate the antibiotics through molecular modifications [56]. In addition, the dormant state of bacteria in biofilms may passively promote tolerance to antimicrobial substances [57]. On the other hand, the proximity of cells within biofilms and the environmental DNA in the EPS structure favor horizontal gene transfer. Accordingly, *C. jejuni* transmits chromosomally encoded antibiotic resistance genes more frequently in biofilms than in a bacterial planktonic manner [58]. In this study, significant down regulation of the biofilm biosynthesis gene and the quorum sensing regulator (*FlaA* and *LuxS*, respectively) are reported after treatment with green tea extract. Castillo et al. [44] found that the epigallocatechin ingredient of green tea could inhibit the movement and biofilm formation of *Campylobacter* species by phenotypic methods, though no molecular method could confirm their results.

Although this in vitro approach is considered an alternative to conventional antimicrobials, it is still ignored by medical practice due to the lack of suitable scientific and clinical evidence. Therefore, numerous in vivo studies should be carried out to assess the efficacy and safety of the medicinal plants in animal and human models. Despite the drawbacks of the in vitro model systems, it may be more suitable than in vivo models for the understanding of the mechanisms of drug-induced toxicity due to their lower structural and functional heterogeneity [59,60]. This is a preliminary in vitro validation of the natural products used to mitigate bacterial resistance. However, there is no way to support their clinical use in the treatment of gastroenteritis due to *Campylobacter* infection without in vivo studies.

## 4. Materials and Methods

### 4.1. Sampling

A total of 400 samples of various sources, comprising chicken (n = 290), the environment (n = 60) and human (n = 50), were collected from Zagazig city, Sharkia governorate, Egypt between September 2021 and December 2023. Chicken samples, including cloacal swabs, cecal parts, breast meat, thigh meat and neck skin (n = 50 each), gizzard, and liver (n = 20 each), were collected from recently slaughtered, apparently healthy broiler chickens from 20 different outlets, with each sample representing one bird. Environmental samples, including water, feed and litter (n = 20 each), were obtained from the same outlets. However, human feces were collected from gastroenteritis patients attending clinical laboratories. Samples were collected in sterile Bolton enrichment broth (Oxoid, Hampshire, UK) and transported to the laboratory within three hours in an ice box for bacteriological analysis. Collection of samples complied with the general guidelines of the Faculty of Veterinary Medicine, Zagazig University, Egypt. The human study was performed according to the World Medical Associates Ethics (Declaration of Helsinki) for studies involving humans. Written informed consent was acquired from the patients participating in the investigation after a full explanation of the purpose of the study.

### 4.2. Isolation and Identification of Thermophilic Campylobacter Species

For the isolation of *Campylobacter* species, samples were incorporated in Bolton enrichment broth then incubated at 41 °C for 24 h in culture vessels with <1 cm and with firmly capped lids. After enrichment, a loopful of broth culture was streaked onto modified Cefoperazone Charcoal Deoxycholate agar (mCCDA, Oxoid, UK) prepared from *Campylobacter* blood-free selective agar base CM0739 and CCDA selective supplement SR0155 (Oxoid, UK). The plates were incubated at 41.5 °C in darkness for 48 h under microaerophilic condition (5% O_2_, 10% CO_2_ and 85% N_2_) using CamyGen sachets (Oxoid, UK) [61]. Presumptive identification of the isolates as *C. jejuni* or *C. coli* was performed adopting biochemical reactions including oxidase, catalase, indoxyl acetate and hippurate hydrolysis as well as their susceptibilities to cephalothin and nalidixic acid (30 µg/disc, each) [62].

### 4.3. Molecular Confirmation of Campylobacter Species

Polymerase chain reaction (PCR) was applied for the confirmation of *Campylobacter* species. DNA extraction was operated using the QIAamp DNA Mini Kit (Qiagen, Germantown, MD, USA) obeying the guidelines provided by the manufacturer. Conventional PCR amplification procedures were performed to detect the *23S rRNA*, *mapA*, and *ceuE* genes of genus *Campylobacter*, *C. jejuni*, and *C. coli*, respectively, using the oligonucleotide primer sets presented in Table S1 [63,64]. All PCR procedures were carried out in triplicate using the Emerald Amp GT PCR Master Mix (Takara, Mountain View, CA, USA), in accordance with the manufacturer`s instructions. The DNAs of *C. jejuni* (NCTC11322) and *C. coli* (NCTC11366) were included in all PCR assays as positive controls whereas PCR grade water was considered a negative control.

### 4.4. Antimicrobial Susceptibility Testing

*Campylobacter* isolates were examined for their susceptibilities to 13 antimicrobials representing different categories adopting the Kirby–Bauer disc diffusion method [65]. The antimicrobial classes included β-lactams [penicillin (P; 10 µg), ampicillin–sulbactam (AMS; 20 µg), amoxicillin–clavulanic acid (AMC; 30 µg) and cefotaxime (CTX; 10 µg)], aminoglycosides [gentamycin (CN; 10 µg)], fluoroquinolones [norfloxacin (NOR; 5 µg) and ciprofloxacin (CIP; 5 µg)], macrolides (erythromycin (E; 15 µg) and tylosin (TL; 15 µg)], phenicols [chloramphenicol (C; 30 µg)], tetracyclines [tetracycline (TE; 30 µg)], polypeptides [colistin (CT; 10 µg)], and sulphonamides [sulfamethoxazole–trimethoprim (SXT (25 µg)] (Oxoid, UK). The interpretive criteria of the Clinical Laboratory Standards Institute [66] and the European committee for antimicrobial susceptibility testing [67] were followed for analysis of the results. The multiple antimicrobial resistance (MAR) indices were estimated as previously documented [68] and MDR (resistance to ≥ three antimicrobial categories) were detected as documented elsewhere [69].

### 4.5. Quantitative Assessment of Biofilm Formation by Campylobacter Species

Induction of biofilm formation by *Campylobacter* isolates was performed in triplicate using flat-bottom 96-well polystyrene microtiter plate (Techno plastic products, Trasadingen, Swizerland) as presented elsewhere [70]. Briefly, 20 µL of 10*^6^* CFU/mL initial bacterial suspension in tryptic soy broth (TSB; Oxoid, UK) was put into each well, then incubated for 48 h at 41.5 °C. The wells were completely aspirated and underwent two rounds of washing with PBS (200 µL, pH 7.2) to remove the planktonic bacteria, they were then airdried for 15 min. Biofilms were stained with 100 µL/well of crystal violet (0.01% *w*/*v*) for 30 min. The wells were then washed twice with PBS and air-dried. After resuspending the stained biomass in an 80:20, *v*/*v* ethanol acetone solution, an ELISA reader (Stat-fax 2100, Foster City, CA, USA) was used to measure the optical density (OD) at 570 nm. Wells with non-inoculated medium and biofilm-producing bacterium were employed, respectively, as positive and negative controls. *Campylobacter* isolates were categorized using the cut-off optical density value (OD cut = OD avg of negative control + 3 × standard deviation (SD) of ODs negative control) based on biofilm formation ability as follows: no biofilm producers (OD ≤ DO cut), weak biofilm producers (OD cut ≤ OD × OD cut), moderate biofilm producer (2 × OD cut, OD ≤ 4 × OD cut) or strong biofilm producers (OD > 4 × OD cut) [71].

### 4.6. Plant Extracts and Their Preparation

Plant extracts of green tea (*Camellia sinensis*), rosemary (*Rosmarinus officinalis*) and ginger (*Zingiber officinale*) were purchased from Makin Company, Egypt. The plant extracts were prepared according to Yam and coauthors [72] with a few modifications. In brief, 200 g of each plant powder was extracted into 500 mL of ethanol (70%) for 2 h. Then, high-speed centrifugation and filtration were used to remove the insoluble particles. The filtrate was reduced to 120 mL in a rotary evaporator then extracted five times with 180 mL of ethyl acetate. The organic phases were collected and concentrated to 25 mL at a rotary evaporator, 25 mL of distilled water was added before the remaining ethyl acetate and aqueous phase were freeze-dried. Five grams of the dried precipitate were dissolved in 100 mL of PBS (pH 7.4), which were used together as a stock solution of the forementioned plant extracts. Finally, two-fold serial dilution of the stock solutions, i.e., 25, 12.5, 6.25, 3.12 and 1.56 mg/mL, were prepared in order to test their antimicrobial activities against MDR campylobacters.

### 4.7. Antimicrobial Activities of the Plant Extracts

The antimicrobial activities of green tea, rosemary and ginger plant extracts were estimated against drug-resistant *Campylobacter* isolates using the standard disc diffusion and broth microdilution techniques [73]. For the disc diffusion assay, 100 µL bacterial suspension of 1.5 × 10^8^ CFU/mL in TSB was cultured in Muller–Hinton agar media (MHA, Oxoid, UK). A sterile Whatman No. 5 filter paper of 6 mm diameter (Machery-Magel, Doren, Germany) was impregnated with 20 µL of each plant extract at various concentrations (50, 25, 12.5, 6.25, 3.12 and 1.56 mg/mL) then placed on the agar culture media and incubated under microaerophilic condition at 41.5 °C for 48 h. The inhibition zone diameters were measured in millimeters, and interpretation of results was reported as documented elsewhere [74]. A sterile filter paper disk soaked in 50 µL of PBS was considered a control negative, whereas an antibiotic disk of gentamycin (10 µg) was also used as a control positive.

The broth microdilution technique was carried out to determine the minimal inhibitory concentrations (MICs) of tested plant extracts against drug-resistant *Campylobacter* isolates using the 96-well culture plates. Two-fold serial dilutions of each plant extract were performed from the stock solution, then 100 µL of each dilution was dispended in 96-well culture plates. Thereafter, 100 µL of bacterial suspension was added to each well and incubated under a microaerophilic environment at 41.5 °C for 1–2 days. The lowest concentration of each plant extract exhibiting no growth was considered as MIC.

### 4.8. Antibiofilm Assay

The activities of natural antimicrobials on mature biofilms performed by *Campylobacter* isolates were studied using the microtiter plate technique as presented elsewhere [75] with modifications. In brief, 20 µL of bacterial suspension (10*^6^* cells/mL) was placed into each well containing 180 µL of TSB. After 24 h of biofilm development, the medium was withdrawn, and each well was washed by sterile PBS. Two hundred TSB microliters, having different concentrations of the green tea extract (SIC, MIC and 2MIC) were added, then incubated for 24 h. The biofilms that developed on the wells’ sides were stained for 15 min with 100 μL of 0.1% crystal violet and then washed again with PBS to remove the excess dye. The stained biofilms were then dissolved with 33% acetic acid for 15 min. The dissolved biofilms were transferred to a new 96-well microtiter plate and then measured using a microplate reader (PowerWave 340, Bio-tek Instruments, Winooski, VT, USA) at 570 nm. The percentage of biofilm inhibition was determined using the following formula:

(A570 of the test/A570 of non-treated control isolate) × 100

where A is the ELISA reader’s absorbance measurement at 570 nm (stat fax 2100, Awareness Technology, Palm City, FL, USA). Both a positive control (wells containing biofilms) and a negative control (a non-biofilm generator) were involved. All procedures were conducted in triplicate.

### 4.9. High-Performance Liquid Chromatography (HPLC) Analysis of Green Tea Extract

Green tea HPLC analysis was carried out using an Agilent 1260 series, Canada. The separation was carried out using Zorbax Eclipse Plus C8 column (4.6 mm × 250 mm, 5 μm). The mobile phase comprised water (A) and 0.05% trifluoroacetic acid in acetonitrile (B) at a flow rate of 0.9 mL/min. The mobile phase was sequentially programmed using the following linear gradient: 0 min (82% A); 0–1 min (82% A); 1–11 min (75% A); 11–18 min (60% A); 18–22 min (82% A); 22–24 min (82% A). The multi-wavelength detector was monitored at 280 nm. The injection volume was 5 μL for each of the sample solutions. The column temperature was maintained at 40 °C [76].

### 4.10. Green Tea Analysis Using Liquid Chromatography–Electrospray Ionization–Tandem Mass Spectrometry (LC-ESI-MS/MS) [77]

#### 4.10.1. Quantitative Analysis of the Total Phenolic and Flavonoid Contents of Green Tea Extract

This was performed using LC-ESI-MS/MS with an ExionLC AC system for separation and a SCIEX Triple Quad 5500+ MS/MS system equipped with an electrospray ionization (ESI) for detection. For the negative ionization mode, the separation was performed using a Poroshell 120 EC-C18 column (3.0 × 100 mm, 2.7 µm). The mobile phases consisted of two eluents; A: 0.1% formic acid in water and B: acetonitrile (LC grade). The mobile phase was programmed as mentioned in Table S2. The injection volume was 5 µL. The negative ionization mode was applied with the following mass spectrometer parameters; curtain gas: 25 psi, ion spray voltage: –4500, and source temperature: 400 °C. Ion source gases 1 and 2 were 55 psi with multiple reaction monitoring (MRM) parameters, as described in Table 8.

#### 4.10.2. Non-Targeted Screening for Catechins (Qualitative Analysis)

The analysis of the sample was performed using LC-ESI-MS/MS with an ExionLC AC system for separation and SCIEX Triple Quad 5500+ MS/MS system equipped with an electrospray ionization (ESI) for detection. For the negative ionization mode, the separation was performed with an Ascentis^®^ Express 90 Å C18 column (2.1 × 150 mm, 2.7 µm). The mobile phases consisted of two eluents; A: 5 mM ammonium formate, pH 8 and B: acetonitrile (LC grade). The mobile phase gradient was programmed as in Table S3. The flow rate was 0.25 mL/min, and the injection volume was 5 µL. For MS/MS analysis, negative ionization mode was applied with a scan (EMS-IDA-EPI) from 100 to 1000 Da for MS1 with the following parameters; curtain gas: 25 psi, ion spray voltage: −4500, source temperature: 500 °C. Ion source gases 1 and 2 were 45 psi and from 50 to 1000 Da for MS2 with a declustering potential of −80 and collision energy: −35.

For the positive ionization mode, the separation was performed with an Ascentis^®^ Express 90 Å C18 column (2.1 × 150 mm, 2.7 µm). The mobile phases consisted of two eluents; A: 5 mM ammonium formate, pH 3 and B: acetonitrile (LC grade). The mobile phase gradient was programmed as depicted in Table S3.

### 4.11. Reverse Transcriptase Quantitative Polymerase Chain Reaction (RT-qPCR)

After biofilm formation, as described above, the strong biofilm producer *Campylobacter* isolates were separately exposed to the SIC of the green tea extract then incubated at 41.5 °C for 48 h. *Campylobacter* biofilms with no treatment were considered controls. Biofilms were precisely harvested then gently rinsed with PBS to remove the non-adherent cells. Total RNA was extracted from biofilms of both treated and untreated *Campylobacter* isolates using aQIAampRN easy Mini kit (Qiagen, Germany) according to the manufacturer’s guidelines. Relative expression of the biofilm genes, *FlaA* and *LuxS* [78,79], were detected by one step RT-qPCR using the QuantiTect SYBR Green RT-PCR kit (Qiagen, Germany) in the MX3005p real-time PCR thermal cycler (Stratagene, Lajolla, CA, USA) according to the manufacturer’s instructions. Each sample was subjected to RT-qPCR in triplicate, and the mean values were used for subsequent analysis. Oligonucleotide primer sequences involved in the RT-qPCR technique are shown in Table S1. Melting curves were created to confirm the amplified products’ specificity (one cycle of 94 °C for 1 min, 50 °C for 1 min, and 94 °C for 1 min for each of the *FlaA* and *LuxS* genes). The relative expression levels of the examined genes were normalized to the constitutive expression of the *23S rRNA* housekeeping gene. Fold changes in the target gene’s transcript levels in treated *Campylobacter* biofilm producers, compared with their levels in untreated producers, were estimated according to the 2^−∆∆CT^ method [80].

### 4.12. Statistical Analysis

The Shapiro–Wilk and Levene tests were employed to confirm both the homogeneity and normality of variances. The occurrence of *Campylobacter* species in animals, alongside antimicrobial resistance, was analyzed using the chi-square test (χ^2^). The inhibitory effects of different concentrations of plant extracts on *Campylobacter* isolates were assessed through the Kruskal–Wallis test. Linear regression was utilized to determine the amount of decrease in the inhibition zone with each unit decrease in the concentration of various plant extracts. To investigate the significant differences in the MICs among different plant extracts, the Tukey’s honestly significant difference (HSD) test was applied. The significant distinctions in the optical density pre- and post-treatment were evaluated using the *t*-test. Fold changes in the expression of examined biofilm genes after treatment of *Campylobacter* isolates with the SICs of green tea extract were examined through a one-way analysis of variance (ANOVA). A *p*-value < 0.05 was considered statistically significant.

## 5. Conclusions

These results provide new perspectives on the antimicrobial possibility of natural herbal products against planktonic *Campylobacter* cells. Additionally, the current study demonstrates the anti-quorum sensing and antibiofilm activities of green tea against MDR biofilm-producing *Campylobacter* isolates using molecular methods. The knowledge gained from this study is an in vitro preliminary validation for the application of green tea extract as a promising therapy for the mitigation of *Campylobacter* resistance in veterinary medicine. However, further in vivo studies should be implemented in the future to confirm its predictions of safety, toxicity, and efficacy.

## Figures and Tables

**Table 1:** Occurrence of *Campylobacter* species in animal, environmental and human sources.

Source (No.)	Sample Type (No.)	Overall Occurrence of *Campylobacter* Isolates No. (%)	*Campylobacter* Species No. (%)		*p*-Value
			*C. jejuni*	*C. coli*	
Chickens and their products (290)	Cloacal swabs (50)	10 (20.00)	1 (2.00)	9 (18.00)	0.0003
	Breast muscles (50)	5 (10.00)	4 (8.00)	1 (2.00)	0.0507
	Thigh muscles (50)	9 (18.00)	6 (12.00)	3 (6.00)	0.1572
	Liver (20)	12 (60.00)	11 (55.00)	1 (5.00)	0.0001
	Gizzard (20)	7 (35.00)	5 (25.00)	2 (10.00)	0.1088
	Neck skin (50)	8 (16.00)	6 (12.00)	2 (4.00)	0.0455
	Cecal parts (50)	15 (30.00)	13 (26.00)	2 (4.33)	0.0001
Total positives		66 (22.76)	45 (15.52)	21 (7.24)	0.0001
Environmental samples (60)	Litter (20)	3 (15.00)	3 (15.00)	0 (0.00)	0.0326
	Feed (20)	1 (5.00)	1 (5.00)	0 (0.00)	0.0862
	Water (20)	1 (5.00)	1 (5.00)	0 (0.00)	0.0862
Total positives		5 (8.33)	5 (8.33)	0 (0.00)	0.0862
Human (50)	Stool (50)	15 (30.00)	10 (20.00)	5 (10.00)	0.0678
Total	400	86 (21.5)	61 (15.25)	25 (6.25)	0.0001

*p*-values were calculated based on chi-square test. *p*-values at 0.05 are statistically significant.

**Table 2:** Antimicrobial resistance of *Campylobacter* species isolated from animal, environmental and human sources.

AMA	*C. jejuni* Isolates (n = 61)								Total	*p*-Value	*C. coli* Isolates (n = 25)					Total	*p*-Value
	Chicken Source (n = 46)				Environmental Source (n = 5)			Human Stool (n = 10)			Chicken Source (n = 20)				Human Stool (n = 5)		
	Muscle (10)	Internal Organs (29)	Neck Skin (6)	Cloacal Swab (1)	Litter (3)	Feed (1)	Water (1)				Muscle (4)	Internal Organ (5)	Nick Skin (2)	Cloacal Swab (9)			
P	9 (90.00)	25 (86.21)	6 (100.00)	1 (100.00)	2 (66.66)	0 (0.00)	1 (100.00)	8 (80.00)	52 (85.25)	0.0001	2 (50.00)	5 (100.00)	2 (100.00)	7 (77.78)	4 (80.00)	20 (80.00)	0.0001
SAM	4 (40.00)	5 (17.24)	3 (50.00)	1 (100.00)	1 (33.33)	0 (0.00)	0 (0.00)	2 (20.00)	16 (26.23)	0.0001	2 (50.00)	2 (40.00)	1 (50.00)	0 (0.00)	2 (40.00)	7 (28.00)	0.0001
AMC	2 (20.00)	6 (20.69)	2 (33.33)	1 (100.00)	1 (33.33)	1 (100.00)	0 (0.00)	5 (50.00)	18 (29.51)	0.0001	1 (25.00)	2 (40.00)	0 (0.00)	1 (11.11)	4 (80.00)	8 (32.00)	0.0001
E	7 (70.00)	24 (82.76)	6 (100.00)	1 (100.00)	2 (66.66)	1 (100.00)	1 (100.00)	7 (70.00)	49 (80.33)	0.5416	2 (50.00)	5 (100.00)	2 (100.00)	9 (100.00)	5 (100.00)	23 (92.00)	0.5153
TL	3 (30.00)	4 (13.79)	2 (33.33)	1 (100.00)	1 (33.33)	1 (100.00)	0 (0.00)	4 (40.00)	16 (26.23)	0.0001	1 (25.00)	1 (20.00)	1 (50.00)	0 (0.00)	2 (40.00)	5 (20.00)	0.0001
CT	7 (70.00)	26 (89.66)	4 (66.67)	1 (100.00)	3 (100.00)	1 (100.00)	1 (100.00)	9 (90.00)	52 (85.25)	0.6129	4 (100.00)	4 (80.00)	2 (100.00)	9 (100.00)	3 (60.00)	22 (88.00)	0.7641
C	4 (40.00)	5 (17.24)	2 (33.33)	1 (100.00)	1 (33.33)	1 (100.00)	1 (100.00)	2 (20.00)	17 (27.87)	0.0001	0 (0.00)	2 (40.00)	1 (50.00)	1 (11.11)	3 (60.00)	7 (28.00)	0.0001
CTX	8 (80.00)	25 (86.21)	6 (100.00)	1 (100.00)	3 (100.00)	0 (0.00)	1 (100.00)	7 (70.00)	51 (83.61)	0.0001	4 (100.00)	5 (100.00)	2 (100.00)	9 (100.00)	5 (100.00)	25 (100.00)	1.000
TE	9 (90.00)	24 (82.76)	4 (66.67)	1 (100.00)	2 (66.66)	1 (100.00)	1 (100.00)	8 (80.00)	50 (81.97)	0.4534	4 (100.00)	5 (100.00)	2 (100.00)	6 (66.67)	4 (80.00)	21 (84.00)	0.5686
CN	3 (30.00)	4 (13.79)	2 (33.33)	0 (0.00)	1 (33.33)	1 (100.00)	0 (0.00)	3 (30.00)	14 (22.95)	0.0001	1 (25.00)	1 (20.00)	1 (50.00)	0 (0.00)	2 (40.00)	5 (20.00)	0.0001
CIP	4 (40.00)	4 (13.79)	2 (33.33)	1 (100.00)	1 (33.33)	1 (100.00)	0 (0.00)	4 (40.00)	17 (27.87)	0.0001	0 (0.00)	2 (40.00)	1 (50.00)	0 (0.00)	2 (40.00)	5 (20.00)	0.0001
NOR	4 (40.00)	6 (20.69)	1 (16.61)	1 (100.00)	1 (33.33)	0 (0.00)	1 (100.00)	3 (30.00)	17 (27.87)	0.0001	1 (25.00)	2 (40.00)	0 (0.00)	0 (0.00)	0 (0.00)	3 (12.00)	0.0001
SXT	3 (30.00)	6 (20.69)	2 (33.33)	1 (100.00)	0 (0.00)	0 (0.00)	1 (100.00)	4 (40.00)	17 (27.87)	0.0001	1 (25.00)	2 (40.00)	1 (50.00)	0 (0.00)	2 (40.00)	6 (24.00)	0.0001

Values represent the number of *Campylobacter* isolates (%); *p*-values were calculated based on chi-square test. *p*-values at 0.05 are statistically significant. AMA, antimicrobial agent; P, penicillin; SAM, ampicillin sulbactam; AMC, amoxicillin–clavulanic acid; E, erythromycin; TL, tylosin; CT, colistin; C, chloramphenicol; CTX, cefotaxime; TE, tetracycline; CN, gentamycin; CIP, ciprofloxacin; NOR, norfloxacin; SXT, sulfamethoxazole–trimethoprim.

**Table 3:** Characterization and biofilm formation by MDR *Campylobacter* isolates.

Isolate No.	Source	*Campylobacter* Species	Antimicrobial Resistance Pattern	MAR Index	Biofilm Formation	
					OD 570 *	Degree
1	Human stool	*C. jejuni*	P, SAM, AMC, E, CIP, NOR, SXT, CN, TE, C, CT, CTX, TL	1.00	0.52	Strong
2	Chicken gizzard	*C. jejuni*	P, SAM, AMC, E, CIP, NOR, SXT, CN, TE, C, CT, CTX, TL	1.00	0.55	Strong
3	Chicken neck skin	*C. jejuni*	P, SAM, AMC, E, CIP, NOR, SXT, CN, TE, C, CT, CTX, TL	1.00	0.63	Strong
4	Chicken liver	*C. jejuni*	P, SAM, AMC, E, CIP, NOR, SXT, CN, C, CT, CTX, TL	0.92	0.13	Weak
5	Human stool	*C. jejuni*	P, SAM, E, CIP, NOR, SXT, CN, TE, CT, CTX, TL	0.85	0.6	Strong
6	Chicken litter	*C. jejuni*	P, SAM, E, CIP, NOR, CN, TE, C, CT, CTX, TL	0.85	0.55	Strong
7	Human stool	*C. coli*	P, SAM, AMC, CIP, NOR, SXT, CN, TE, CT, CTX, TL	0.85	0.48	Strong
8	Chicken liver	*C. jejuni*	P, SAM, CIP, NOR, SXT, CN, TE, C, CT, CTX, TL	0.85	0.49	Strong
9	Chicken breast muscle	*C. coli*	P, SAM, AMC, E, NOR, SXT, CN, TE, CT, CTX, TL	0.85	0.29	Moderate
10	Chicken neck skin	*C. jejuni*	P, SAM, AMC, E, CIP, SXT, CN, C, CT, CTX, TE	0.85	0.50	Strong
11	Chicken neck skin	*C. coli*	P, SAM, E, CIP, SXT, CN, TE, C, CT, CTX, TL	0.85	0.21	Moderate
12	Chicken thigh muscle	*C. jejuni*	P, SAM, E, CIP, NOR, SXT, TE, C, CT, CTX, TL	0.85	0.59	Strong
13	Chicken thigh muscle	*C. jejuni*	P, SAM, E, CIP, NOR, CN, TE, C, CT, CTX, TL	0.85	0.20	Moderate
14	Chicken cecal swab	*C. jejuni*	P, SAM, AMC, E, CIP, NOR, TE, C, CT, CTX, TL	0.85	0.49	Strong
15	Chicken cecal part	*C. jejuni*	P, SAM, AMC, E, CIP, NOR, SXT, CN, TE, CT, TL	0.85	0.24	Moderate
16	Chicken cecal part	*C. coli*	P, SAM, AMC, E, CIP, NOR, SXT, TE, C, CTX, TL	0.85	0.44	Strong
17	Chicken breast muscle	*C. jejuni*	P, E, CIP, NOR, SXT, CN, TE, C, CTX, TL	0.77	0.20	Moderate
18	Chicken breast muscle	*C. jejuni*	P, SAM, AMC, E, CIP, SXT, TE, C, CT, CTX	0.77	0.60	Strong
19	Feed	*C. jejuni*	P, E, CIP, CN, TE, C, CT, TL	0.62	0.61	Strong
20	Water	*C. jejuni*	P, NOR, SXT, TE, C, CT, CTX	0.54	0.62	Strong

P, penicillin; SAM, ampicillin–sulbactam; AMC, amoxicillin–clavulanic acid; E, erythromycin; CIP, ciprofloxacin; NOR, norfloxacin; SXT, sulfamethoxazole–trimethoprim; CN, gentamycin; TE, tetracycline; C, chloramphenicol; CT, colistin; CTX, cefotaxime; TL, tylosin; MAR, multiple antibiotic resistance; OD, optical density. * The data represents ELISA reading at OD 570 nm.

**Table 4:** Antibacterial activities of various plant extracts against *Campylobacter* isolates.

Isolate No.	*Campylobacter* Species	Plant Extract																					*p*-Value
		Green Tea							Rosemary							Ginger							
		Disc Diffusion (mm)						MIC (mg/mL)	Disc Diffusion (mm)						MIC (mg/mL)	Disc Diffusion (mm)						MIC (mg/mL)	
		50	25	12.5	6.25	3.12	1.56		50	25	12.5	6.25	3.12	1.56		50	25	12.5	6.25	3.12	1.56		
1	*C. jejuni*	37	32	22	19	10	5	3.12 ^a^	15	11	5	2	0	0	25 ^a^	20	16	12	6	4	0	12.5 ^b^	0.0281
2	*C. jejuni*	36	31	25	20	11	8	1.56 ^a^	16	11	5	3	0	0	25 ^a^	19	13	10	5	0	0	12.5 ^b^	0.0108
3	*C. jejuni*	37	30	25	19	10	5	3.12 ^a^	14	7	5	0	0	0	50 ^a^	20	15	10	7	3	0	12.5 ^b^	0.0148
4	*C. jejuni*	37	30	24	20	10	5	3.12 ^a^	15	10	3	0	0	0	25 ^a^	14	11	8	4	0	0	12.5 ^b^	0.0084
5	*C. jejuni*	38	33	25	20	10	5	3.12 ^a^	15	10	4	2	0	0	25 ^a^	19	12	7	3	0	0	25 ^a^	0.0130
6	*C. jejuni*	37	30	23	18	8	4	3.12 ^a^	15	11	6	3	0	0	25 ^a^	20	17	9	5	0	0	12.5 ^b^	0.0469
7	*C. coli*	38	30	25	20	9	4	3.12 ^a^	12	9	5	0	0	0	25 ^a^	18.	15	11	7	4	0	12.5 ^b^	0.0155
8	*C. jejuni*	35	30	25	20	9	3	3.12 ^a^	15	11	4	1	0	0	25 ^a^	15	10	7	4	0	0	25 ^a^	0.0139
9	*C. coli*	26	29	24	19	11	8	1.56 ^a^	18	10	4	0	0	0	25 ^a^	19	15	10	6	3	0	12.5 ^b^	0.0161
10	*C. jejuni*	36	31	24	20	12	8	1.56 ^a^	15	11	4	0	0	0	25 ^a^	17	12	6	4	0	0	25 ^a^	0.0052
11	*C. coli*	36	31	23	16	9	4	3.12 ^a^	16	10	6	3	0	0	25 ^a^	19.	15	9	6	0	0	12.5 ^b^	0.0416
12	*C. jejuni*	37	30	22	17	8	4	3.12 ^a^	16	10	3	0	0	0	25 ^a^	19	13	6	3	0	0	25 ^a^	0.0311
13	*C. jejuni*	38	32	23	18	11	8	1.56 ^a^	16	11	5	2	0	0	25 ^a^	19	16	10	7	4	0	12.5 ^b^	0.0167
14	*C. jejuni*	37	32	23	18	9	4	3.12 ^a^	15	10	4	0	0	0	25 ^a^	18	13	9	5	0	0	12.5 ^b^	0.0227
15	*C. jejuni*	35	30	23	19	8	3	3.12 ^a^	15	10	4	0	0	0	25 ^a^	18	14	9	5	0	0	12.5 ^b^	0.0295
16	*C. coli*	37	30	23	19	9	5	3.12 ^a^	16	10	5	0	0	0	25 ^a^	19	15	10	5	0	0	12.5 ^b^	0.0267
17	*C. jejuni*	35	32	23	18	10.	4	3.12 ^a^	16	10	3	0	0	0	25 ^a^	19	15	10	6	2	0	12.5 ^b^	0.0254
18	*C. jejuni*	37	32	24	19	9.	3	3.12 ^a^	15	11	5	3	0	0	25 ^a^	19	16	9	5	0	0	12.5 ^b^	0.0345
19	*C. jejuni*	35	32	22	18	9	4	3.12 ^a^	15	10	3	0	0	0	25 ^a^	20	15	10	6	3	0	12.5 ^b^	0.0289
20	*C. jejuni*	37	29	24	21	9	5	3.12 ^a^	16	11	5	2	0	0	25 ^a^	19	15	11	6	3	0	12.5 ^b^	0.0255
Overall average		36.0 ± 0.57	30.8 ± 0.25	23.6 ± 0.23	18.9 ± 0.27	9.55 ± 0.24	4.95 ± 0.38	*p* = 0.0175 b-reg = 0.576	15.3 ± 0.25	10.2 ± 0.21	4.4 ± 0.21	1.05 ± 0.28	0 ± 0.00	0 ± 0.00	*p* = 0.0005 b-reg = 0.334	18.5 ± 1.25	14.1 ± 0.35	9.15 ± 0.41	5.25 ± 0.36	1.3 ± 0.27	0 ± 0.00	*p* = 0.0048 b-reg = 0.370	

a, b Means with different superscripts in the same row are significantly different; b-reg, b regression; MIC, minimum inhibitory concentration.

**Table 5:** HPLC analysis results showing the constituents of green tea extract.

Constituents	Area	Concentration (µg/mL)
Gallic acid	641.83	424.69
Chlorogenic acid	47.59	54.64
Catechin	77.77	150.77
Methyl gallate	0.00	0.00
Coffeic acid	78.38	52.68
Syringic acid	25.79	14.67
Rutin	129.69	176.50
Ellagic acid	82.75	56.96
Coumaric acid	0.00	0.00
Vanillin	0.00	0.00
Ferulic acid	0.00	0.00
Naringenin	0.00	0.00
Rosmarinic acid	0.00	0.00
Daidzein	0.00	0.00
Querectin	0.00	0.00
Cinnamic acid	40.74	6.24
Kaempferol	0.00	0.00
Hesperetin	0.00	0.00

**Table 6:** Total phenolic and flavonoid compounds of the green tea extract analyzed by LC-ESI-MS/MS.

Component Name	Expected RT	Area *	RT	Conc. (µg/mL)
Gallic acid 168.9/124.9	1.67	8.34 × 10^7^	1.69	86.51
Caffeic acid 178/135	5.83	1.57× 10^6^	5.88	0.5761
Rutin 609/299.9	9.13	5.36 × 10 ^7^	9.15	19.42
Coumaric acid 162.9/119	7.7	1.97 × 10 ^7^	7.73	1.747
Naringenin 271/151	20.98	6.17 × 10 ^5^	20.94	47.01
Querectin 301/151	18.16	2.24 × 10 ^6^	18.14	0.4713
Ellagic acid 301/145	8.97	2.30 × 10 ^5^	9.01	0.7675
3.4-Dihydroxybenzoic acid 152.9/109	3.13	5.98 × 10 ^6^	3.19	5.9
Hesperetin 301/136	22.62	N/A	N/A	ND
Cinnamic acid 146.9/77	18.16	N/A	N/A	ND
Methyl gallate 183/124	5.04	1.57 × 10 ^6^	5.11	ND
Kaempferol 284.7/93	22.08	5.46 × 10 ^5^	22.06	0.1809
Ferulic acid 192.8/133.9	8.89	1.84 × 10 ^5^	8.94	0.324
Syringic acid 196.8/181.9	6.23	6.13 × 10 ^4^	6.3	0.3735
Apigenin 269/151	21.47	N/A	N/A	ND
Catechin 288.8/109	5.05	1.68 × 10 ^6^	5.11	6.688
Daidzein 253/132	16.22	N/A	N/A	ND
Chlorogenic acid 353/191	5.1	6.63 × 10 ^6^	5.16	1.628
Resveratrol 227/185	14.27	4.00 × 10 ^4^	14.36	0.1487
Rosmarinic acid 359.1/161	13.42	N/A	N/A	ND

Rt, retention time; N/A, non-applicable; ND, not detected. * The scientific notation could be converted into decimal notation using the following website: https://converthere.com/numbers/2.3e+7-written-out (accessed on 2 December 2024).

**Table 7:** Antibiofilm activity of green tea extract against MDR-biofilm-producing *Campylobacter* isolates.

Isolate No.	*Campylobacter* Species	OD Pre-Treatment	OD Post Treatment (Reduction %) *	*p*-Value
1	*C.* *jejuni*	0.52	0.14 (73.1)	0.0001
2	*C.* *jejuni*	0.55	0.25 (54.6)	0.0001
3	*C.* *jejuni*	0.63	0.21 (66.7)	0.0001
5	*C.* *jejuni*	0.6	0.21 (65.0)	0.0001
6	*C.* *jejuni*	0.55	0.14 (74.6)	0.0001
7	*C. coli*	0.48	0.10 (79.2)	0.0001
8	*C.* *jejuni*	0.49	0.13 (73.5)	0.0001
10	*C.* *jejuni*	0.50	0.15 (70.0)	0.0001
12	*C.* *jejuni*	0.59	0.13 (79.0)	0.0001
14	*C.* *jejuni*	0.49	0.12 (75.5)	0.0001
16	*C. coli*	0.44	0.22 (50.0)	0.0001
18	*C.* *jejuni*	0.60	0.22 (63.3)	0.0001
19	*C.* *jejuni*	0.61	0.14 (77.1)	0.0001
20	*C.* *jejuni*	0.62	0.24 (61.29)	0.0001
Overall average		0.547 ± 0.01 ^a^	0.171 ± 0.01 ^b^	0.0001

OD, optical density. * Numbers in between brackets indicate reduction % in biofilm formation by each tested *Campylobacter* isolate. a, b Means with different superscripts in the same row are significantly different (*p* < 0.05).

**Table 8:** Summary of optimized parameters for the quantitative analysis of the phenolic and flavonoid contents of green tea.

ID	Q1 (*m*/*z*)	Q3 (*m*/*z*)	RT (min)	CE (V)	CXP (V)	DP (V)
Gallic acid 168.9/124.9	168.9	124.9	1.67	−30	−11	−110
Gallic acid 168.9/79	168.9	79	1.67	−30	−11	−110
Caffeic acid 178/135	178.9	135	5.83	−22	−9	−115
Caffeic acid 178/107	178.9	107	5.83	−30	−7	−115
Rutin 609/299.9	609	299.9	9.13	−48	−15	−230
Rutin 609/270.9	609	270.9	9.13	−70	−9	−230
Coumaric acid 162.9/119	162.9	119	7.7	−20	−7	−90
Coumaric acid 162.9/93	162.9	93	7.7	−40	−5	−90
Vanillin 151/136	151	136	7.48	−12	−9	−140
Vanillin 151/92	151	92	7.48	−16	−7	−140
Naringenin 271/151	271	151	20.98	−24	−25	−130
Naringenin 271/119	271	119	20.98	−34	−11	−130
Querectin 301/151	301	151	18.16	−28	−9	−50
Querectin 301/178.8	301	178.8	18.16	−20	−7	−50
Ellagic acid 301/145	301	145	8.97	−40	−14	−120
Ellagic acid 301/245	301	245	8.97	−38	−14	−120
3.4-Dihydroxybenzoic acid 152.9/109	152.9	109	3.13	−40	−5	−75
3,4-Dihydroxybenzoic acid 152.9/90.9	152.9	90.9	3.13	−20	−7	−75
Hesperetin 301/164	301	164	22.62	−23	−10	−125
Hesperetin 301/136	301	136	22.62	−38	−10	−125
Cinnamic acid 146.9/102.6	146.9	102.6	18.16	−17	−6	−60
Cinnamic acid 146.9/77	146.9	77	18.16	−33	−6	−60
Methyl gallate 183/124	183	124	5.04	−30	−10	−110
Methyl gallate 183/140	183	140	5.04	−30	−10	−110
Kaempferol 284.7/93	284.7	93	22.08	−46	−10	−120
Kaempferol 284.7/116.8	284.7	116.8	22.08	−52	−10	−120
Ferulic acid 192.8/133.9	192.8	133.9	8.89	−16	−5	−25
Ferulic acid 192.8/177.9	192.8	177.9	8.89	−12	−5	−25
Syringic acid 196.9/122.8	196.9	122.8	6.23	−24	−5	−30
Syringic acid 196.8/181.9	196.9	181.9	6.23	−12	−5	−30
Apigenin 269/151	269	151	21.47	−15	−7	−35
Apigenin 269/117	269	117	21.47	−15	−7	−35
Catechin 288.8/244.9	288.8	244.9	5.05	−16	−8	−40
Catechin 288.8/109	288.8	109	5.05	−32	−8	−40
Luteolin 284.7/132.9	284.7	132.9	18.14	−38	−10	−50
Luteolin 284.7/150.9	284.7	150.9	18.14	−26	−10	−50
Daidzein 253/132	253	132	16.22	−55	−10	−65
Daidzein 253/91	253	91	16.22	−50	−13	−65
Chlorogenic acid 353/191	353	191	5.1	−23	−10	−60
Chlorogenic acid 353/179	353	179	5.1	−35	−10	−60
Resveratrol227/185	277	187	14.27	−53	−26	−10
Resvertrol 277/143	277	143	14.27	−53	−40	−10
Romarinic acid 359.1/161	359.1	161.030	13.42	−60	−20	−10
Rosmarinic acid 359.1/197	359.1	197.050	13.42	−60	−20	−10

CE, collision energy; CXP, collision cell exit potential. RT, retention time; DP, declustering potential.

## Data Availability

The data presented in this study are available on request from the corresponding author.

## Supplementary Materials

The following supporting information can be downloaded at: https://www.mdpi.com/article/10.3390/antibiotics14010061/s1, Table S1: Oligonucleotide primers used in the study; Table S2: The mobile phase for quantitative analysis of the total phenolic and flavonoid contents of *Camellia sinensis*; Table S3: The mobile phase used for non-targeted screening for catechins (qualitative analysis).
