# Supplementary material for: Green Tea Extract (Theaceae; Camellia sinensis L.): A Promising Antimicrobial, Anti-Quorum Sensing and Antibiofilm Candidate Against Multidrug-Resistant Campylobacter Species
Source: Antibiotics (Basel). 2025 Jan 9;14(1):61. doi: 10.3390/antibiotics14010061 (PMC11760471; doi:10.3390/antibiotics14010061)
Supplement: Supplementary file 1 [file antibiotics-14-00061-s001.zip › antibiotics-3379965-supplementary.pdf]

Table S1. Oligonucleotide primers used in the study.

Target genes	Primers sequences 5 → 3`	Specificity	Annealing temperature (° C)	Product size (bp)	References
23S rRNA	F: TATACCGGTAAGGAGTGCTGGAG<br>R: ATCAATTAACCTTCGAGCACCG	*Campylobacter* species and a housekeeping gene	52	650	[63]
mapA	F: CTATTTTATTTTTGAGTGCTTGTG<br>R: GCTTTATTTGCCATTTGTTTTATTA	*C. jejuni*	58	589	[64]
ceuE	F: AATTGA AAATTG CTCCAACTATG<br>R: TGATTT TATTATTTGTAGCAGCG	*C. coli*	58	462	
FlaA	F:TCCAAATCGGCGCAAGTTCA<br>R:TCAGCCAAAGCTCCAAGTCC	Flagellin	55	855	[78]
LuxS	F:AGTGTTGCAAAAGCTTGGGA<br>R:GCATTGCACAAGTTCCGCAT	Quorum sensing	55	800	[79]

F, forward; R, reverse; Bp base pair.

Table S2: The mobile phase for quantitative analysis of the total phenolic and flavonoid contents of *Camellia sinensis*

Time	%B	Flow rate (mL/min)
0	8	0.5
1	8	0.5
4	15	0.4
12	20	0.4
20	30	0.4
25	45	0.4
25.01	8	0.5
28	8	0.5

Table S3: The mobile phase used for non-targeted screening for catechins (qualitative analysis)

Time	A. Concentration	B. Concentration
	90.0	10.0
1.00	90.0	10.0
30.00	10.0	90.0
35.00	10.0	90.0
35.01	90.0	10.0
40.00	90.0	10.0
40.10	0.0	100.0
50.00	0.0	100.0
